# The unexpected diagnosis of karyomegalic interstitial nephritis in a presumed case of Mesoamerican Nephropathy: a case report

**DOI:** 10.3389/fmed.2025.1465783

**Published:** 2025-03-05

**Authors:** Lawrence Kwon, Jennifer Griffiths, Lanny T. DiFranza

**Affiliations:** ^1^Department of Nephrology, Westchester Medical Center Advanced Physician Services, Mid-Hudson Regional Hospital, Poughkeepsie, NY, United States; ^2^Department of Pathology, Montefiore Medical Center, Bronx, NY, United States

**Keywords:** karyomegalic interstitial nephritis, Mesoamerican Nephropathy, chronic kidney disease of unknown etiology, tubulointerstitial nephropathy, kidney biopsy diagnosis

## Abstract

**Background:**

Chronic kidney disease of unknown etiology (CKDu) is a form of chronic kidney disease commonly found in certain rural populations globally. This condition is characterized by chronic tubulointerstitial nephropathy, yet it lacks specific signature lesions and is believed to have a multifactorial etiology, often associated with environmental toxins. Karyomegalic Interstitial Nephritis (KIN), although a rare form of chronic interstitial nephropathy leading to end-stage kidney disease, is not classified under CKDu.

**Case presentation:**

In this case report, we explore the diagnostic journey of a 40-year-old male farmer from Guatemala. He presented with headache, fever, and facial pain, but laboratory tests revealed significant kidney impairment and liver dysfunction. The pivotal point in his diagnostic workup was a kidney biopsy, which showed severe chronic tubulointerstitial scarring and enlarged, hyperchromatic nuclei in the tubular epithelial cells, confirming KIN. This diagnosis marked a departure from the initial suspicion of Mesoamerican Nephropathy (MEN).

**Conclusion:**

This case underscores the critical need for a comprehensive evaluation in atypical presentations of chronic kidney disease, particularly emphasizing the importance of being vigilant for KIN in areas where MEN is commonly diagnosed.

## Background

Karyomegalic Interstitial Nephritis (KIN) is a rare and often under-recognized cause of chronic kidney disease, characterized by distinct histopathological features. This case report describes a 40-year-old male from Guatemala, initially suspected to have Mesoamerican Nephropathy (MEN), a tubulointerstitial kidney disease common in Central American agricultural communities. The turning point in the diagnostic process came with a kidney biopsy, which unexpectedly revealed findings indicative of KIN, a condition seldom reported in this region.

The patient’s presentation with non-specific systemic symptoms, alongside a significant personal history of heavy alcohol use and a familial history of kidney failure, posed a complex diagnostic puzzle. The case was further complicated by an unusual and initially baffling finding in the urine microscopy, which added another layer of complexity to the differential diagnoses. Ultimately, it was the kidney biopsy that was elucidative, providing a definitive diagnosis of KIN.

This case highlights the diagnostic complexities encountered in regions with overlapping environmental and occupational risk factors for kidney disease. It emphasizes the necessity of a broad diagnostic approach, including consideration of rare conditions like KIN, in patients presenting with kidney dysfunction.

## Case presentation

A 40-year-old male farmer from Guatemala presented with headache, fever, and facial pain. He had consumed ~10 cans of beer per weekend for >20 years, but had been sober for the past 6 months. His mother, who also worked as a farmer and laborer, died from complications of kidney failure and an unspecified malignancy.

The patients’ health had declined for the past year, with associated fatigue, exertional chest pain, dyspnea, nocturia, back pain, and loss of appetite, resulting in a weight loss of >20 lbs. In his last 2 months in Guatemala before relocating to the US, he developed daily fevers, which he treated with acetaminophen and NSAIDs. One month before admission, he was hospitalized with *Streptococcus pneumoniae* meningitis with bacteremia.

**Figure 1:**
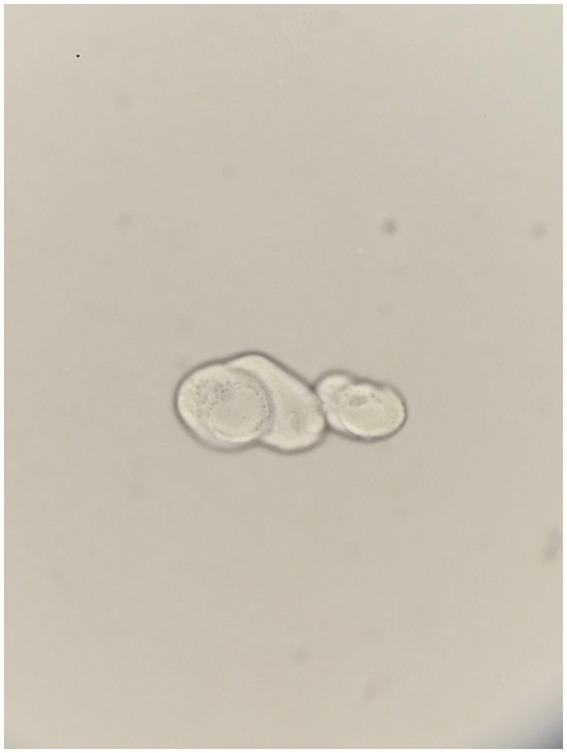
Atypical cells in unstained urine, arranged linearly, with two cells displaying notably large and vesicular nuclei. Original magnification, 800x.

Physical examination of the patient revealed a blood pressure of 102/59 mmHg, a weight of 55 kg, a BMI of 23 kg/m^2^, and mildly icteric sclerae. Detailed laboratory findings are presented in Table 1. Kidney ultrasound demonstrated atrophic kidneys with increased echogenicity, measuring 7.2 × 4.5 × 3.6 cm for the right kidney, and 6.1 × 3.1 × 3.7 cm for the left. Abdominal CT and ultrasound both indicated the presence of perihepatic ascites and splenomegaly, measuring 14.5 cm, while the gallbladder appeared normal. Microscopic examination of the urine revealed cells with abnormally large nuclei (Figure 1).

**Table 1:** Laboratory findings.

Parameter	Value	Normal range
Hematology		
WBC (x 10^9^/1)	2.47	4.0–11.0
Hgb (g/dL)	8.9	13.8–17.2 (male)
MCV (fl)	104.5	80–100
Pit (x 10^9^/1)	173	150–450
Neut (%)	45	40–60
Eos (%)	21	1–4
Baso (%)	4	<1
Chemistry		
BUN (mg/dL)	80	7–20
sCr (mg/dL)	8.25	0.84–1.21
Na (mmol/L)	131	135–145
K (mmol/L)	5.2	3.5–5.1
Cl (mmol/L)	100	98–107
CO2 (mmol/L)	19	23–29
UA (mg/dL)	12.4	3.4–7.0
Phos (mg/dL)	7.9	2.5–4.5
Ca (mg/dL)	8.5	8.6–10.2
PTH (pg/mL)	185	15–65
Vit D (ng/mL)	<12.5	30–100
HbA1C (%)	4	<5.7
Liver function tests		
T.Bili (mg/dL)	4	0.1–1.2
AST (UL)	100	10–40
ALT (U/L)	95	7–56
ALP (U/L)	1,071	40–129
Alb (g/dL)	3.6	3.4–5.4
NH3 (gmol/L)	32	15–45
Urinalysis		
RBCs	None	None
WBCs	None	None
uProt/uCr (g/g)	1.3	<0.2
Albumin (%)	7	–
FENa (%)	16.5	–
FEIJA (%)	39.1	–
Serologies		
ANA	Negative	Negative
ANCA	Negative	Negative
Anti-dsDNA	Negative	Negative
ASO (Todd units)	400	<200
Complement levels		
C3 (mg/dL)	74	90–180
C4 (mg/dL)	12	10–40
Comp (mg/dL)	28	41–90

WBC, White Blood Cell count; Hgb, Hemoglobin; MCV, Mean Corpuscular Volume; Plt, Platelet Count; Neut, Neutrophils; Eos, Eosinophils; Baso, Basophils; BUN, Blood Urea Nitrogen; sCr, Serum Creatinine; Na, Sodium; K, Potassium; Cl, Chloride; CO2, Carbon Dioxide; UA, Uric Acid; Phos, Phosphorus; Ca, Calcium; PTH, Parathyroid Hormone; Vit D, Vitamin D; HbA1C, Hemoglobin A1C; T.Bili, Total Bilirubin; AST, Aspartate Aminotransferase; ALT, Alanine Aminotransferase; ALP, Alkaline Phosphatase; Alb, Albumin; NH3, Ammonia; uProt/uCr, Urine Protein-to-Creatinine Ratio; FENa, Fractional Excretion of Sodium; FEUA, Fractional Excretion of Uric Acid; ANA, Antinuclear Antibody; ANCA, Anti-Neutrophil Cytoplasmic Antibodies; Anti-dsDNA, Anti-double stranded DNA antibodies; ASO, Anti-Streptolysin O; C3, Complement Component 3; C4, Complement Component 4; Comp, Total Complement.

The suspected diagnosis was MEN, considering the patient’s young age and lack of clinical hypertension or diabetes. He resided and worked as a farmer in Guatemala, facing frequent heat stress, dehydration, occupational exposure to toxic agrochemicals, contaminated food and water, and/or aerosolized toxins. Differential diagnoses included hepatorenal syndrome and infection-related glomerulonephritis. The two large nucleated cells in the urine were unexplained.

His liver dysfunction was presumed to be ethanol-induced; ceruloplasmin, anti-smooth muscle, and anti-mitochondrial tests were negative. An MRI of the abdomen without contrast revealed a normal-sized liver with typical morphology and no evidence of fat accumulation or suspicious lesions. Normal iron studies ruled out iron overload. Genetic testing for hemochromatosis gene variants (C282Y and H63D) was negative.

Ascitic fluid of 1.5 liters was removed, containing 153 WBCs and < 2,000 RBCs/mL, albumin 1.8 g/dL, and total protein 3.2 g/dL. Differential showed 28% polynucleated cells, 35% lymphocytes, 19% macrophages, 5% mesothelial cells, and 13% monocytes. Cultures were negative. Serum-ascites albumin gradient was 1.8 g/dL, consistent with portal hypertension.

**Figure 2:**
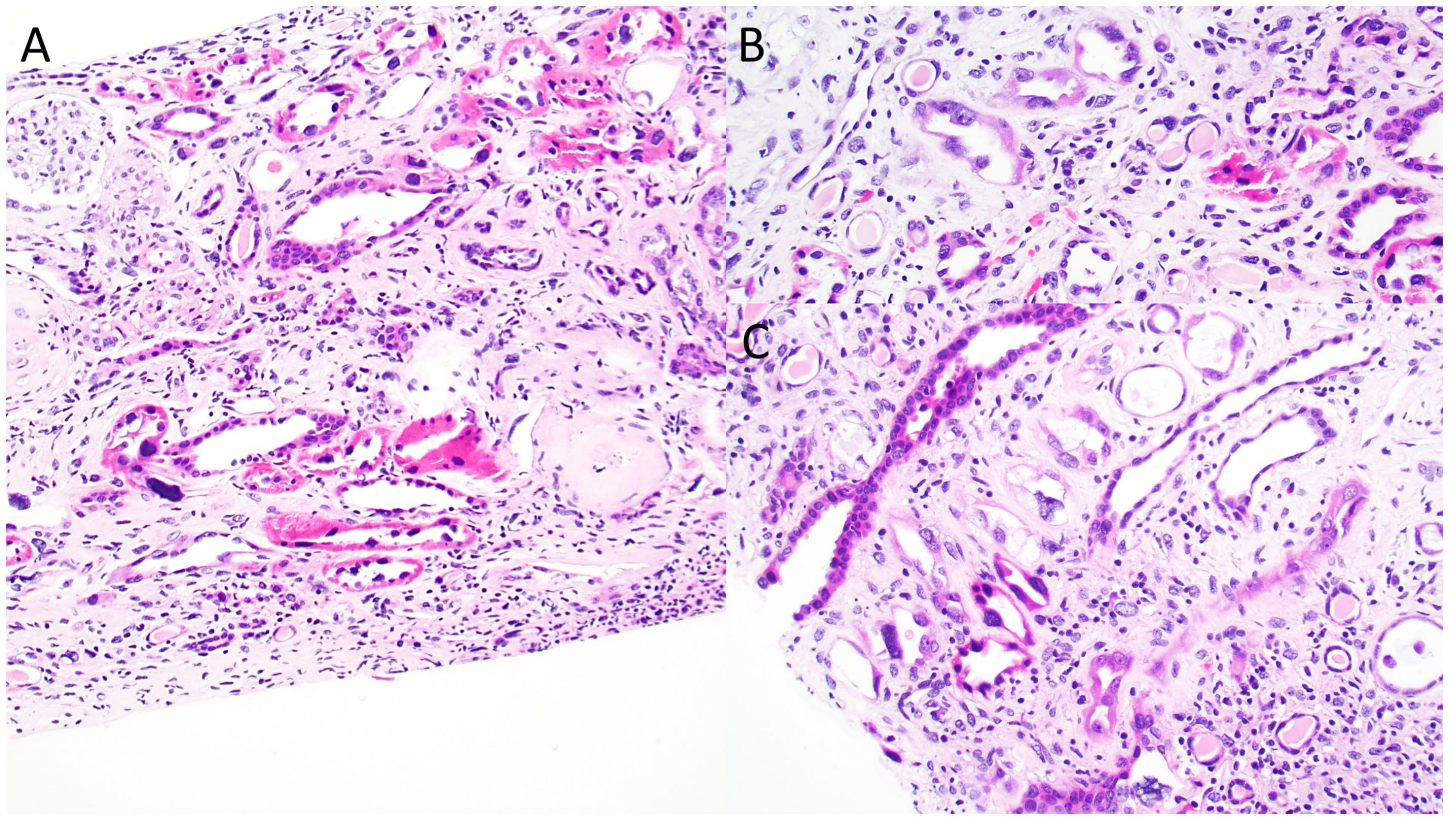
Photomicrographs from the kidney biopsy depicting bizarre nuclear enlargement in tubular epithelial cells. Images highlight changes of acute tubular injury, including simplification of the tubular epithelial lining and individual epithelial cell necrosis. **(A)** Hematoxylin & Eosin, original magnification, 40x; **(B)** Hematoxylin & Eosin, original magnification, 100x.

**Figure 3:**
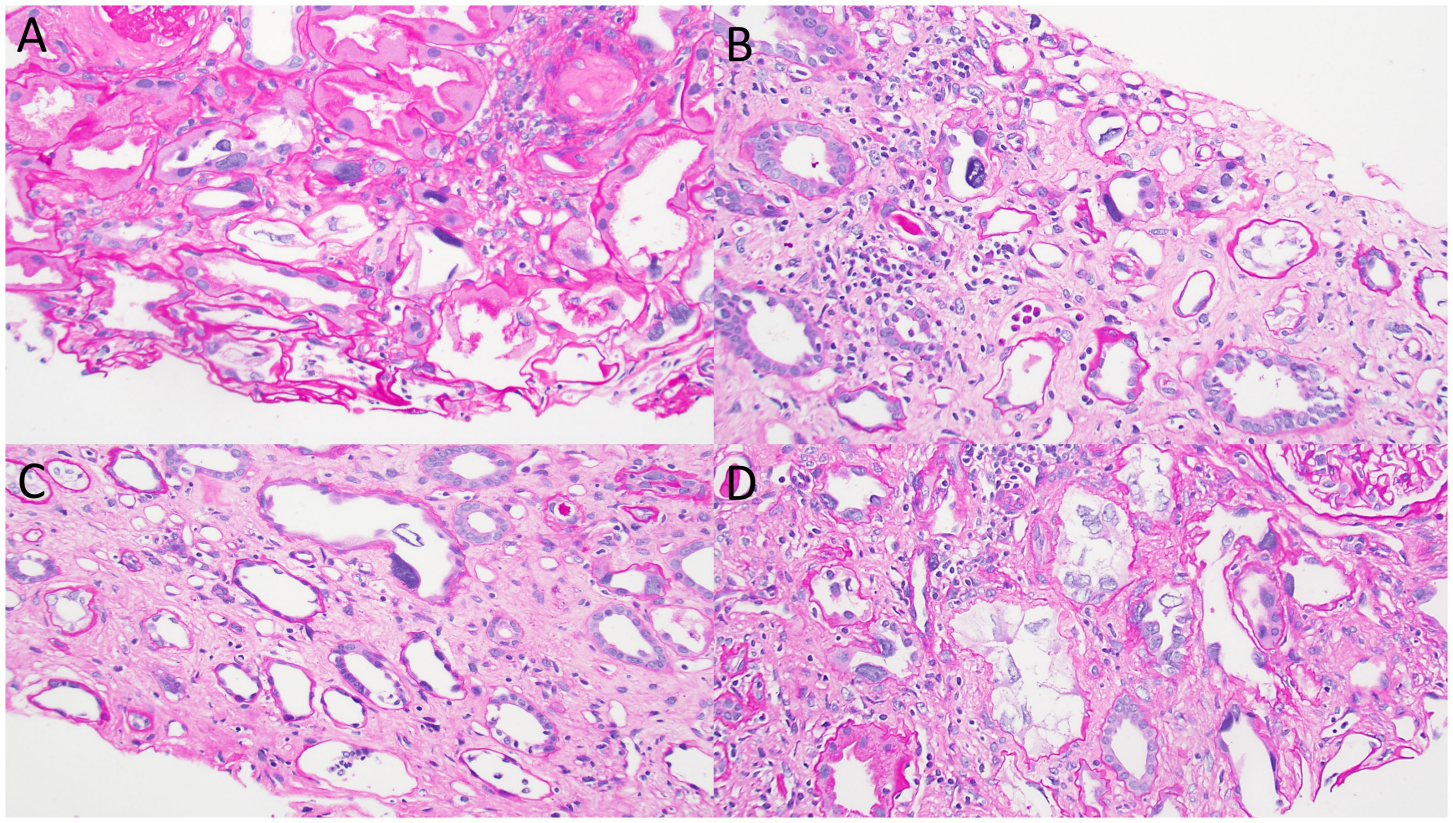
Photomicrographs from the kidney biopsy showing bizarre nuclear enlargement in tubular epithelial cells. These images illustrate acute tubular injury features, including simplification of the tubular epithelial lining and individual epithelial cell necrosis. **(A-D)** Periodic acid-Schiff, original magnification, 100x for all images.

**Figure 4:**
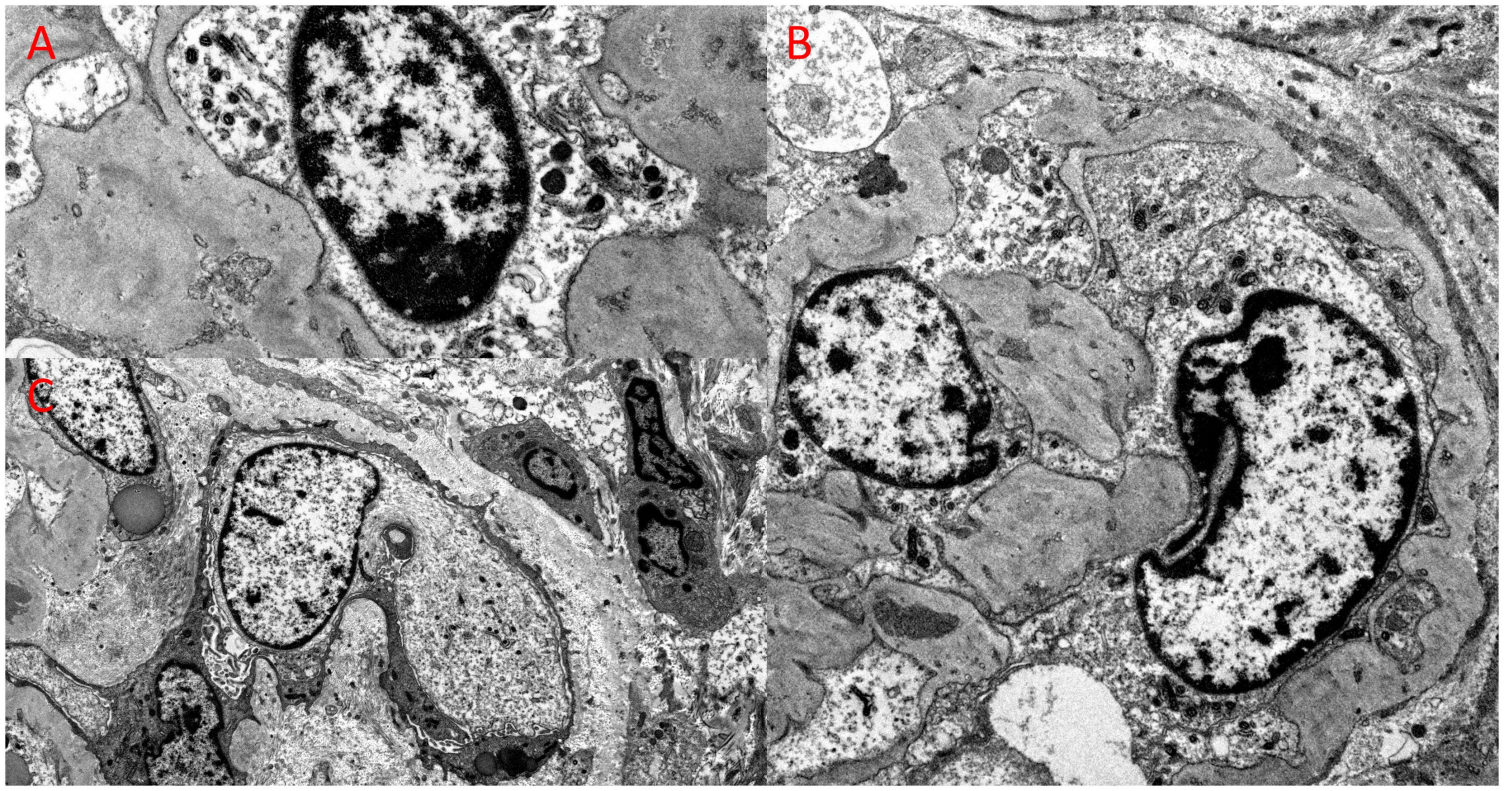
Ultrastructural examination from the kidney biopsy, revealing bizarre nuclear enlargement in glomerular endothelial and epithelial cells. **(A)** Electron microscopy, original magnification, 26,000x; **(B)** Electron microscopy, original magnification, 10,000x; **(C)** Electron microscopy, original magnification, 12,000x.

Kidney biopsy (Figures 2–4) showed 141/184 sampled glomeruli to be globally sclerotic. Remaining glomeruli showed diffuse ischemic changes, characterized by global thickening and wrinkling of glomerular basement membranes, and partial retraction of the glomerular tuft. Mesangial areas were unremarkable. No significant endocapillary hypercellularity was evident. Podocytes appeared focally swollen. Severe tubulointerstitial scarring involved ~60–70% of the sampled cortex, with associated moderate interstitial inflammation. Proximal and distal tubules showed diffuse, bizarre enlargement of nuclei, attenuation of brush borders, simplification of lining epithelium, sloughing of epithelial cells into lumina, and occasional intraluminal granular epithelial cell casts. Nuclei of tubular epithelial cells were predominantly hyperchromatic, but some exhibited hypochromasia. Hyperchromatic nuclei appeared smudged; others showed avesicular chromatin pattern. Many nuclei were enlarged by 4-5x. Nuclear changes were present in both proximal and distal tubules. Arteries lacked significant intimal fibrosis, but displayed focal nuclear enlargement of medial myocytes. Arterioles showed mild mural hyalinosis. An immunohistochemical stain for cytomegalovirus was negative.

Genetic testing was performed using a next-generation sequencing–based 385 kidney gene panel (Renasight™ test by Natera, Inc., Austin, TX). Informed consent was obtained prior to saliva swab collection. Results revealed a homozygous FAN1 c.2916 + 2 T > G (p.?) variant, located on chromosome 15 (GRCh37: g.31222876 T > G; NM_014967.5: c.2916 + 2 T > G). This variant disrupts the canonical splice donor site for exon 13, altering the highly conserved consensus sequence necessary for proper splicing. Multiple splice prediction tools predict this variant abolishes canonical splice activity, likely leading to aberrant splicing and altered gene function. To our knowledge, this splicing variant has not been previously reported in the literature. Loss-of-function variants in FAN1 have been implicated in karyomegalic interstitial nephritis (PubMed: 22772369) and associated with hereditary nonpolyposis colorectal cancer (PubMed: 26052075). This variant is absent from the Broad gnomAD dataset.

The patient is currently undergoing hemodialysis three times weekly.

## Discussion

This case presented a fascinating diagnostic challenge, given the multitude of potential differential diagnoses. Initially, the leading contender was MEN, a tubulointerstitial disease demonstrating features aligned with the patient’s clinical profile; he fit classic MEN risk factors: a slender, young male farmer from Central America with no hypertension or diabetes, a history of pesticide and agrochemical exposure, extensive NSAID use, hyperuricemia, and familial history of advanced CKD (1). In MEN, proteinuria is often <1 g/24 h (1).

Another diagnostic consideration was acute kidney Injury (AKI) on chronic kidney disease – hepatorenal syndrome (CKD-HRS). AKI-HRS seemed improbable due to proteinuria >500 mg/g (2), an abnormal kidney ultrasound indicative of parenchymal disease (2), and a FENa of 16.5%, contrasting with the typically low FENa (< 1%) of AKI-HRS (3).

Despite the patient’s recent *Streptococcal* infection, elevated anti-streptolysin O values, and hypocomplementemia, infection-related glomerulonephritis was ruled out, due to the absence of hematuria or other nephritic symptoms (4). The depressed complement was attributed to chronic liver disease (5), while lupus nephritis was rendered unlikely by negative autoimmune serologies.

KIN is a rare hereditary condition linked to mutations in the FAN1 gene, which encodes an essential nuclease involved in the Fanconi anemia DNA damage response pathway. The prevalence is less than 1 per 1,000,000.^1^ Fewer than 100 cases have been documented in the literature (6, 7). KIN typically causes slowly progressive chronic kidney disease that can lead to end-stage kidney failure by early adulthood. There are no specific therapies (6, 7).

KIN must be suspected clinically because it can be diagnosed only with kidney biopsy and has no characteristic clinical signs or symptoms or unique laboratory findings. Patients typically have mild proteinuria, usually <1 g/day, and glucosuria. Fewer than one-third have urinary sediment abnormalities, predominantly hematuria (7). However, one published case had a urinary sediment showing 3 atypical cells with abnormally large nuclei (8). Approximately half of patients have a history of recurrent upper respiratory tract infections and abnormal liver function tests. Karyomegaly is not confined to the kidneys, and has been demonstrated in other organs, such as the liver, lung, brain, and skin (7).

Kidney biopsy findings typically consist of severe tubulointerstitial scarring along with nonspecific glomerulosclerosis. The disease’s key feature is abnormally enlarged and hyperchromatic nuclei in karyomegalic tubular epithelial cells that line the proximal and distal tubules (7).

In FAN1-deficient kidney tubular epithelial cells, abnormal DNA re-replication occurs during the prior S-phase of the cell replication cycle, leading to karyomegaly. This condition arises through irregular polyploidization triggered by DNA re-replication, with increased p21 expression and cell-cycle arrest at G2. Blocking p21 could potentially halt upregulation of replication licensing factors and prevent nuclear enlargement, indicating that p21 may represent a therapeutic target for ameliorating KIN-associated kidney damage (9).

FAN1 may be more specifically engaged in the repair of a possible “kidney-specific” DNA damage. Godin et al. (10) reported 2 related cases of KIN associated with a homozygous FAN1 mutation that displayed detectable ochratoxin A in urine and blood (10). Exposure to ochratoxin A has been identified in other patients with KIN, suggesting that FAN1 mutations could render tubular cells susceptible to environmental genotoxin-induced DNA damage (10).

KIN has additionally been linked with side effects of immune checkpoint inhibitors containing the vedotin molecule, including brentuximab (11), enfortumab (12), and the alkylating agent ifosfamide (13).

This case report is unique because another rare disorder, MEN, confounded the differential diagnosis. The similarity in presentation and pathologic findings between the two conditions is intriguing.

To the best of our knowledge, this is only the second report in the literature (8) showing associated karyomegalic changes on urinary cytology. Such cellular abnormalities are commonly seen in conditions like urothelial carcinoma (14), and with the cytopathic effects of BK virus (15). However, these are not typical findings MEN.

KIN and MEN show some similar findings on kidney biopsy, with both diseases presenting with predominant tubulointerstitial scarring without significant glomerular inflammation or vascular sclerosis. The presence of chronic glomerular ischemia, as indicated by wrinkling of glomerular capillaries, has been reported in both conditions (16). However, KIN typically manifests with karyomegalic tubular epithelial cells, a feature absent in MEN (16).

## Conclusion

We have found no detailed reports of KIN in Central America. Due to limited diagnostics and similar presentation to MEN, incidence of KIN in this region may be underreported. As KIN is also linked to exposure to toxic agents (17), further study of this region may yield valuable insights. Central America’s unique environmental context could reveal novel insights. Such investigation is vital, considering that the region’s specific industrial and agricultural exposures may contribute to both KIN and MEN. There may be genetic or environmental triggers specific to the region. Gaining a proper understanding of these associations is essential for advancing our understanding of KIN’s pathogenesis, and may facilitate the development of innovative treatment modalities.

## Data Availability

The original contributions presented in the study are included in the article/supplementary material, further inquiries can be directed to the corresponding author.

## Footnotes

^1^ orpha.net
